# Brain-Specific Rescue of *Clock* Reveals System-Driven Transcriptional Rhythms in Peripheral Tissue

**DOI:** 10.1371/journal.pgen.1002835

**Published:** 2012-07-26

**Authors:** Michael E. Hughes, Hee-Kyung Hong, Jason L. Chong, Alejandra A. Indacochea, Samuel S. Lee, Michael Han, Joseph S. Takahashi, John B. Hogenesch

**Affiliations:** 1 Department of Cellular and Molecular Physiology, Yale School of Medicine, New Haven, Connecticut, United States of America; 2 Center for Sleep and Circadian Biology, Northwestern University, Evanston, Illinois, United States of America; 3 Departments of Neurobiology and Physiology, Northwestern University, Evanston, Illinois, United States of America; 4 Laboratory of Malaria and Vector Research, National Institute of Allergy and Infectious Diseases, National Institutes of Health, Bethesda, Maryland, United States of America; 5 Department of Neuroscience, University of Texas Southwestern Medical Center, Dallas, Texas, United States of America; 6 Howard Hughes Medical Institute, University of Texas Southwestern Medical Center, Dallas, Texas, United States of America; 7 Department of Pharmacology, Institute for Translational Medicine and Therapeutics, University of Pennsylvania School of Medicine, Philadelphia, Pennsylvania, United States of America; Charité–Universitätsmedizin Berlin, Germany

## Abstract

The circadian regulatory network is organized in a hierarchical fashion, with a central oscillator in the suprachiasmatic nuclei (SCN) orchestrating circadian oscillations in peripheral tissues. The nature of the relationship between central and peripheral oscillators, however, is poorly understood. We used the tetOFF expression system to specifically restore *Clock* function in the brains of *Clock^Δ19^* mice, which have compromised circadian clocks. Rescued mice showed normal locomotor rhythms in constant darkness, with activity period lengths approximating wildtype controls. We used microarray analysis to assess whether brain-specific rescue of circadian rhythmicity was sufficient to restore circadian transcriptional output in the liver. Compared to *Clock* mutants, *Clock*-rescue mice showed significantly larger numbers of cycling transcripts with appropriate phase and period lengths, including many components of the core circadian oscillator. This indicates that the SCN oscillator overcomes local circadian defects and signals directly to the molecular clock. Interestingly, the vast majority of core clock genes in liver were responsive to *Clock* expression in the SCN, suggesting that core clock genes in peripheral tissues are intrinsically sensitive to SCN cues. Nevertheless, most circadian output in the liver was absent or severely low-amplitude in *Clock*-rescue animals, demonstrating that the majority of peripheral transcriptional rhythms depend on a fully functional local circadian oscillator. We identified several new system-driven rhythmic genes in the liver, including *Alas1* and *Mfsd2*. Finally, we show that 12-hour transcriptional rhythms (i.e., circadian “harmonics") are disrupted by *Clock* loss-of-function. Brain-specific rescue of *Clock* converted 12-hour rhythms into 24-hour rhythms, suggesting that signaling via the central circadian oscillator is required to generate one of the two daily peaks of expression. Based on these data, we conclude that 12-hour rhythms are driven by interactions between central and peripheral circadian oscillators.

## Author Summary

Circadian rhythms confer adaptive advantage by allowing organisms to anticipate daily rhythms in their environment, such as light and temperature cycles. In mammals, the central circadian time-keeping mechanism resides within the hypothalamus, integrating information from the retina and synchronizing circadian oscillations in peripheral tissues, such as the liver. Nevertheless, the nature of this regulatory relationship is not completely understood. Here we use mice with disrupted circadian rhythmicity stemming from a mutation in *Clock*, a key component of the circadian time-keeping mechanism. Using recently developed transgenic technology, we expressed normal CLOCK protein in the brain, resulting in mice with normal brain rhythmicity, but defective circadian rhythmicity everywhere else. Using microarrays to detect transcriptional rhythms in the livers of these mice, we found that 95 genes showed normal oscillations (albeit with generally lower amplitudes), even in the presence of a defective liver circadian clock. This observation suggests that many rhythmic genes in peripheral tissues are driven directly from the brain. Finally, we showed that harmonics of circadian oscillations, e.g. 12-hour rhythms, revert to 24-hour rhythms in these mice, indicating that both liver and brain-derived cues drive different aspects of these rhythms.

## Introduction

Circadian rhythms are daily oscillations of behavior and physiology that allow organisms to anticipate and respond to predictable daily changes in their environment [1]–[4]. In animals, these environmental variables include light, temperature, food availability, and predation. As a consequence, circadian rhythms regulate behaviors such as feeding rhythms and sleep/wake cycles [2]. At a tissue and cellular level, circadian rhythms compartmentalize the activity of biochemical pathways to appropriate times of day in tissues throughout the body [1]. Taken as a whole, the circadian regulatory network significantly influences normal organismal physiology, and contributes to the pathogenesis of clinically significant conditions including cancer, heart disease, and metabolic disorders [5]–[8].

Self-sustained circadian oscillations are generated at a molecular level via an elaborate transcriptional/translational feedback loop [9]. The positive arm of this feedback loop is mediated by bHLH-PAS transcription factors, BMAL1 and CLOCK/NPAS2 [10], [11], which heterodimerize and drive the expression of downstream target genes. Among these target genes are *Period (Per)* and *Cryptochrome (Cry)*, whose protein products accumulate in the cytoplasm, associate with each other, and ultimately translocate to the nucleus. Once in the nucleus, PER and CRY inhibit BMAL1/CLOCK activity, repressing their own transcription and thus forming the negative arm of the circadian oscillator [9]. In parallel, a second feedback loop is generated via RORE binding activators (*Rora*, *Rorb*, *Rorc*) and repressors (*Rev-erb-alpha*,*Rev-erb-beta*), whose transcription is driven by BMAL1/CLOCK [12].

In conjunction with accessory genes that regulate the stability and activity of key circadian proteins [9], these feedback loops comprise the circadian clock. Ultimately, these molecular oscillations drive 24-hour rhythms of transcription in downstream target genes. Termed “circadian output genes", these transcriptional rhythms are not necessary for sustaining the core circadian oscillator, but are required for mediating circadian regulation of physiology and behavior [13].

Both core circadian oscillations and rhythmic output genes are found in tissues throughout the body [13], [14]. However, not every tissue is equally important for maintaining proper circadian rhythmicity at an organismal level. Rhythms in peripheral tissues, such as liver and skeletal muscle, are self-sustaining *in vitro*, but require inputs from the central circadian oscillator in the suprachiasmatic nuclei (SCN) in the hypothalamus for proper coordination in intact animals [15]. The nature of the regulatory signals between the SCN and peripheral tissues (as well as regulatory cues between peripheral tissues) is poorly understood, but thought to involve neuronal circuitry, humoral factors (e.g. glucocorticoids), and cascades of behavior (i.e. the impact of the sleep wake cycle on eating and elimination).

The general mechanism of circadian oscillations and the genes required for their maintenance is largely conserved between different tissues and species [3], [13], [16], [17]. Nevertheless, circadian output genes are tissue specific, as one would expect given the diverse physiologies regulated by the clock [18]–[20]. Consequently, considerable efforts have been made to characterize circadian transcriptional output at a genome level using microarray technologies [18]–[33]. These studies have made considerable progress towards understanding how tissue-level circadian oscillations are translated into rhythms of organismal physiology. At the same time, this systems-level approach can be used in conjunction with tissue-specific manipulation of gene expression to dissect the relationship between central and peripheral oscillators.

For example, a recent study used liver-specific over-expression of *REV-ERB-alpha* to knock-down *BMAL1* expression in the liver, thereby ablating the local circadian oscillator in an otherwise wildtype animal [21]. They examined the impact of this on circadian regulation of liver gene expression by microarray analysis. This analysis showed that the vast majority of circadian output requires a functional circadian oscillator within the liver. Interestingly, there were several exceptions to this rule, indicating that some circadian genes are driven by systemic cues rather than the local circadian clock. Those genes, including *Per2*, are therefore strong candidates to function as the relay between the SCN and peripheral tissues [21]. Thus, this study established that a functional liver oscillator is required for normal circadian regulation of liver gene expression.

In this manuscript, we seek to extend this research by examining the contribution of central circadian clock function on peripheral physiology. To do this, we employed a tet-OFF expression system to specifically rescue wildtype CLOCK expression in the brains of *Clock*-mutant mice [21], [34]–[36]. Brain-specific *Clock* rescue restored normal behavioral rhythmicity in constant conditions with approximately wildtype period lengths. We used genome-wide transcriptional profiling every two hours for two full days followed by JTK_CYCLE analysis to assess transcriptional circadian output in the mouse liver [37]. The majority of rhythmic genes in the liver required a fully functional liver circadian clock; however, 95 genes still oscillate with circadian periods in the livers of brain-rescued mice, albeit with diminished amplitudes in most cases. We observe that 12-hour transcriptional rhythms (i.e., circadian ‘harmonics’ [19]) are entirely lost in *Clock*-mutant background. Interestingly, brain-specific rescue of *Clock* restores 24-, but not 12-hour rhythmicity to these genes, suggesting that systemic and locally-derived circadian cues are independently required for different peaks of these 12-hour rhythms.

## Results

Inducible, brain-specific expression of wildtype *Clock* was achieved using the tet-OFF system [34]–[36], [38]. Wildtype *Clock* was tagged with an HA epitope and linked to a *tTA*-responsive *tetO* promoter (*tetO::Clock-HA*). At the same time, *tTA* was expressed under the control of the *Secretogranin II* (*Scg2*) promoter (*Scg2::tTA*) which drives expression specifically in the brain, pituitary, and adrenals, with especially high expression in the SCN [39]. When both transgenes were present in the same animal, wildtype *Clock* was expressed at constitutively high levels in the SCN [36]. When these mice were treated with low-doses of Doxycycline (Dox) via their drinking water, the binding of *tTA* to *tetO* promoters was inhibited, and *Clock* expression was abolished [36].

These mice were then crossed into a *Clock^Δ19^* background. *Clock^Δ19^* is an ENU-generated allele of Clock which results in the loss of exon 19 from mature Clock transcripts [40], [41]. *Clock^Δ19^* acts as a dominant-negative by binding to BMAL1 and inhibiting its activity [41]. Consequently, *Clock^Δ19^* animals have severely disrupted circadian behavioral rhythms, with extremely long period lengths and arrhythmicity in prolonged constant conditions [40], [42]–[44].

**Figure 1:**
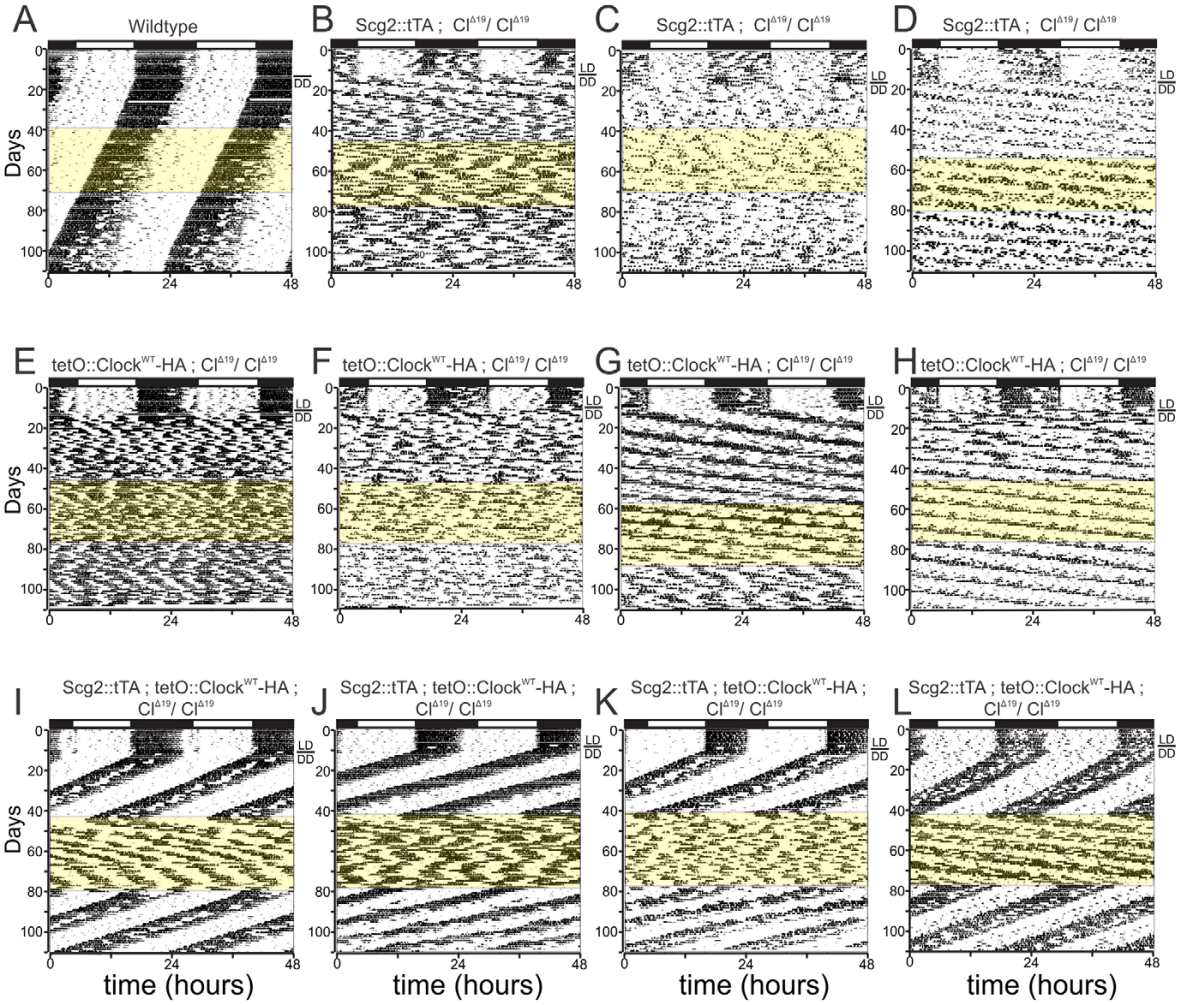
Reversible restoration of circadian rhythm in brain-rescued *Clock* mutant mice. Representative actograms of (A) wildtype mice, control littermates (B, C and D) *Scg2::tTA; Cl^Δ19^/Cl^Δ19^*, (E, F, G and H) *tetO::Clock^WT^; Cl^Δ19^/Cl^Δ19^*, and (I, J, K and L) brain-rescued *Clock* mice (*Scg2*::tTA;tetO::*Clock^WT^*; *Cl^Δ19^/Cl^Δ19^*) on 10 µg/ml Dox treatment. Activity records were double plotted so that 48 hours is represented horizontally, with each day presented beneath and to the right of the preceding day. Wheel-running activity is indicated by black markings. The initial light cycle is depicted at the top of the record. All animals were maintained on a 12-hour light/12-hour dark cycle (LD) for the first 10 to 15 days shown and then transferred to constant darkness (DD), as indicated by the bar next to each record. The yellow shading denotes the time of Dox administration in DD. Brain-rescued *Clock* mice (I, J, K, and L) show restoration of free-running period in DD. When the mice are treated with Dox, brain-rescued *Clock* mice display loss of rhythmicity, similar to that of *Clock* mutant mice (*Cl^Δ19^/Cl^Δ19^*). A water washout returns the mice back to their rhythmic state. Control littermates (A–H) display no circadian rhythmicity in DD. Dox treatment has no effect on activity rhythm in control littermates or wildtype mice (A–H).

Wildtype animals showed robust circadian oscillations in 12-hour light/12-hour dark (LD) conditions with most locomotor activity restricted to the dark phase. The mice maintained these rhythms in constant darkness (DD), with period lengths slightly shorter than 24-hours, in agreement with previous studies (Figure 1A). When either *Scg2::tTA* or *tetO::Clock-HA* were expressed by themselves in a *Clock^Δ19^* background, the mice showed normal LD activity rhythms, but quickly became arrhythmic in DD or had extremely long period lengths (Figure 1B–1H). In all three control genetic backgrounds, the addition of Dox to the drinking water (highlighted in yellow), did not change the circadian behavior of these mice (Figure 1A–1H).

Combining *Scg2:tTA* and *tetO::Clock-HA* in the *Clock^Δ19^* background resulted in mice with normal LD rhythmicity. Unlike their littermate controls (i.e., Figure 1B–1H), however, these mice showed robust circadian rhythmicity in constant conditions (Figure 1I–1L). Previous studies have shown [45] that over-expression of wildtype *Clock* is sufficient to rescue the behavioral phenotype of *Clock^Δ19^*. In agreement with these studies, we detected a modest decrease in the average period length of these animals compared to wildtype (Figure 1A and 1I–1L).

When the rescued mice were treated with Dox (thus inactivating the wildtype *Clock* transgene expression), normal circadian oscillations were quickly lost (Figure 1I–1L). We observed significant animal-to-animal variability in the severity of the resulting phenotype. Generally, however, these mice were either arrhythmic or showed extremely long period lengths while being treated with Dox, consistent with the expected phenotype of *Clock^Δ19^* mice (Figure 1I–1L). This phenotype was completely reversible; when Dox was removed from the drinking water (thus restoring *Clock* transgene expression), normal circadian rhythmicity was quickly reestablished (Table 1). Based on these behavioral data and the previously published expression pattern of *Scg2::tTA ; tetO::Clock-HA* mice [36], we conclude that this system permits the brain-specific rescue of circadian rhythmicity in locomotor activity in a conditional and reversible manner.

**Table 1:** *Clock* expression via the tet-OFF system reversibly rescues normal circadian rhythmicity in DD (+/−standard deviation).

Treatment	Period Length	Number Rhythmic	Number Arrhythmic
Before Dox Treatment	22.6 hours (+/−0.3)	14	0
During Dox Treatment	27.1 hours (+/−0.8)	4	10
After Dox Treatment	22.2 hours (+/−0.5)	14	0

Although brain-specific rescue of *Clock* is sufficient to restore normal behavioral rhythms, it was unclear whether transcriptional and metabolic rhythms in peripheral tissues would be similarly rescued. To answer this, we collected liver samples from wildtype animals as well as *Scg2:tTA ; tetO::Clock-HA* mice that were treated with either normal drinking water or Dox (hereafter referred to as *tetO::Clock* H2O and *tetO::Clock* DOX). Based on the behavioral data presented above, we expected the H_2_O-treated animals (i.e., *Clock* transgene-expressing) to have normal brain rhythmicity and behavioral rhythms, while the Dox-treated animals (i.e., *Clock*-defective) would have disrupted rhythms. It is important to note that in both cases the *Scg2* promoter does not express in the liver, and thus, this peripheral clock is presumed to be *Clock*-defective.

**Figure 2:**
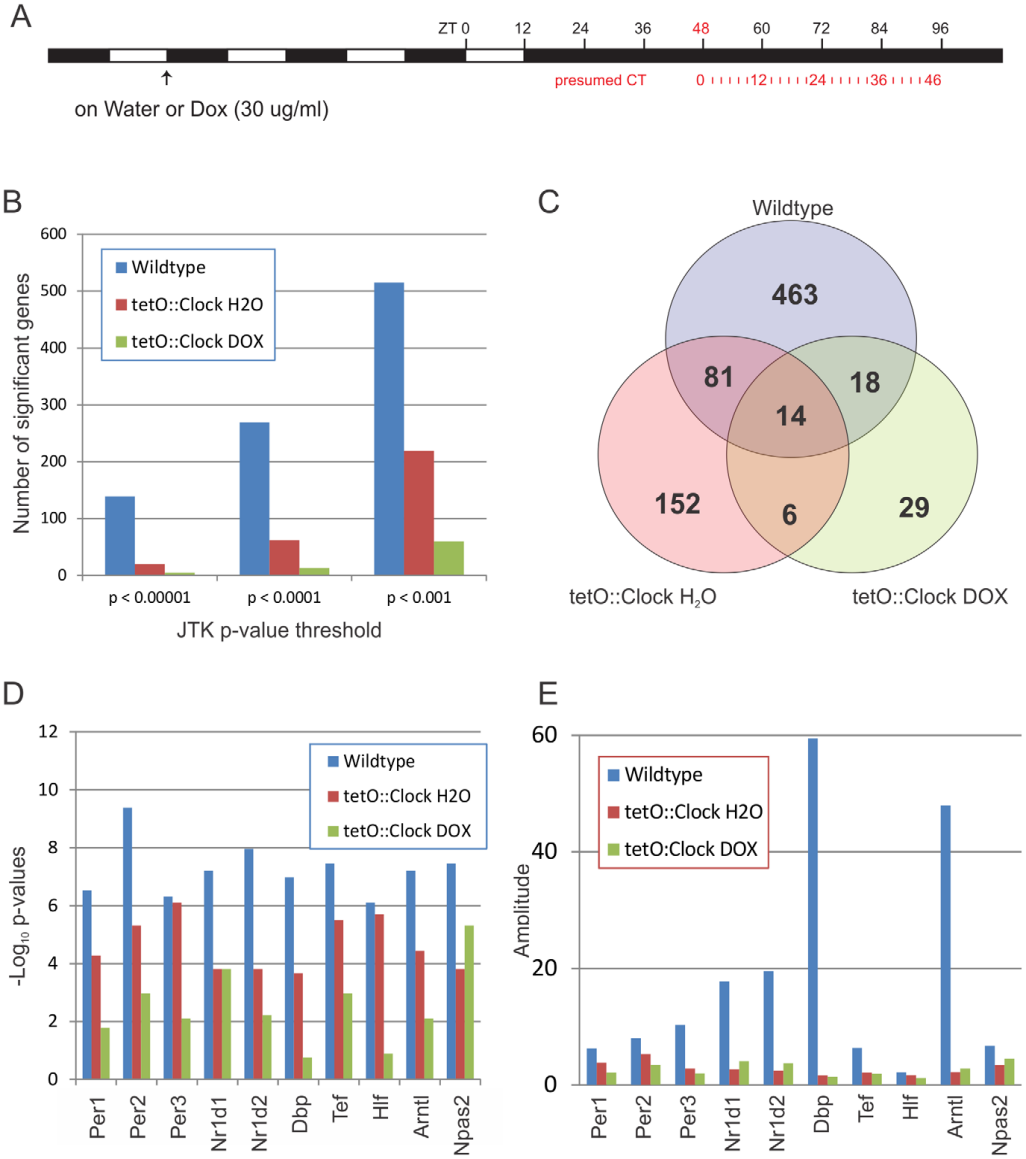
Brain-specific rescue of *Clock* partially restores transcriptional circadian output in the liver. (A) Wildtype and *Clock*-expressing mice (*Scg2::tTA;tetO::Clock^WT^; Cl^Δ19^/Cl^Δ19^*) were entrained to a 12 h∶12 h light∶dark (LD) cycle and then treated with either H_2_O or doxycycline (Dox, 30 µg/ml) 3 days before releasing the mice to constant darkness. Liver samples were collected every two hours for two complete days, starting 48 hours after the final lights-on. (B–E) Cycling genes were detected in microarray datasets using JTK_Cycle. At every p-value threshold tested (B), wildtype livers show considerably more cycling genes than *Clock*-mutant mice (*tetO::Clock* DOX), while the *Clock*-rescue animals (*tetO::Clock* H2O) have an intermediate number of rhythmic genes. Panel (C) shows the number of cycling genes overlapping between the different genotypes/treatments at a p-value threshold of p<0.0011 (corresponding to q<0.05 in wildtype, all period lengths between 10 and 40 hours). The strength of rhythmicity of core clock genes and key circadian output genes (as measured by −Log_10_ p-values) is generally higher in *Clock*-rescue animals (*tetO::Clock* H2O) versus the *Clock*-mutants (*tetO::Clock* DOX). (D) However, *Clock*-rescue (*tetO::Clock* H2O) does not restore wildtype-levels of circadian amplitude in most of these genes (E).

Liver samples were collected every two hours for 48 hours in constant darkness (Figure 2A). Total RNA was extracted and global gene expression was profiled using Affymetrix Mouse Exon Arrays. Cycling genes were detected using JTK_Cycle with false-discovery rates (FDRs) based on the Benjamini-Hochberg procedure [37]. We found 576 cycling genes in wildtype mice at a FDR cutoff of q<0.05 (corresponding to a p-value threshold of p<0.0011), which is consistent with expected levels of transcriptional oscillations given the different sampling resolutions of these studies [18], [19], [46]. Over half of the oscillating genes in this study were previously identified as rhythmic [19], which is an encouraging level of overlap given the typically low agreement between circadian microarray studies [47], [48]. Known core clock genes – including *Per2*, *Bmal1*, and *Rev-erb-alpha* – showed high-amplitude oscillations with expected phase differences, as measured by microarray and confirmed by quantitative PCR (qPCR) (Figure S1). Taken together, these data indicate that this microarray study accurately reflects the underlying circadian transcriptome.

At every statistical threshold we examined, wildtype livers showed significantly more rhythmic transcripts than Dox-treated *tetO::Clock* mice (i.e., *Clock*-mutant). Heatmaps of all cycling genes detected, as well as histograms of their amplitudes are shown in Figure S2. H_2_O-treated (i.e., *Clock*-rescue) mice had an intermediate, partially-rescued phenotype, with considerably more cycling genes detected than Dox-treated animals, though still less than wildtype (Figure 2B and 2C). This pattern was also seen in core clock genes and high-amplitude circadian output genes. The strength of rhythmicity (as measured by the statistical confidence of their detection) consistently demonstrated that *Clock*-rescue (*tetO::Clock* H2O) mice had an intermediate phenotype between wildtype and *Clock*-mutant mice (Figure 2D and Table S1). Brain-specific expression of *Clock* did not rescue the amplitude of most high-amplitude transcriptional rhythms (Figure 2E and Figures S1 and S2). Even though *Clock*-rescue restored a considerable portion of the normal circadian output of the liver, these rhythms were frequently low-amplitude relative to wildtype, indicating that the local circadian oscillator – and in particular, wildtype *Clock* expression – is essential for generating high-amplitude rhythms.

At the statistical threshold we have selected, there are 187 genes that cycle in one or both of the tetO::*Clock* samples without cycling in wildtype. Of these 187 genes, 151 were analyzed in Hughes et al. 2009 [19], and 102 of them were found to oscillate (Table S2). Based on this, we conclude that the majority of non-wildtype cycling genes in the present study are actually bona fide cyclers that did not meet significance given the relatively stringent statistical threshold used. Consistent with this idea, the median p-value for these 102 genes in wildtype samples in the present study is ∼0.1. Given the false-discovery rates for *Clock*-rescue and *Clock*-mutant animals (q<0.10 and q<0.37, respectively at p<0.0011), we conclude that cycling genes specific to non-wildtype backgrounds are rare, in agreement with Kornmann et al. 2007 [21].

**Figure 3:**
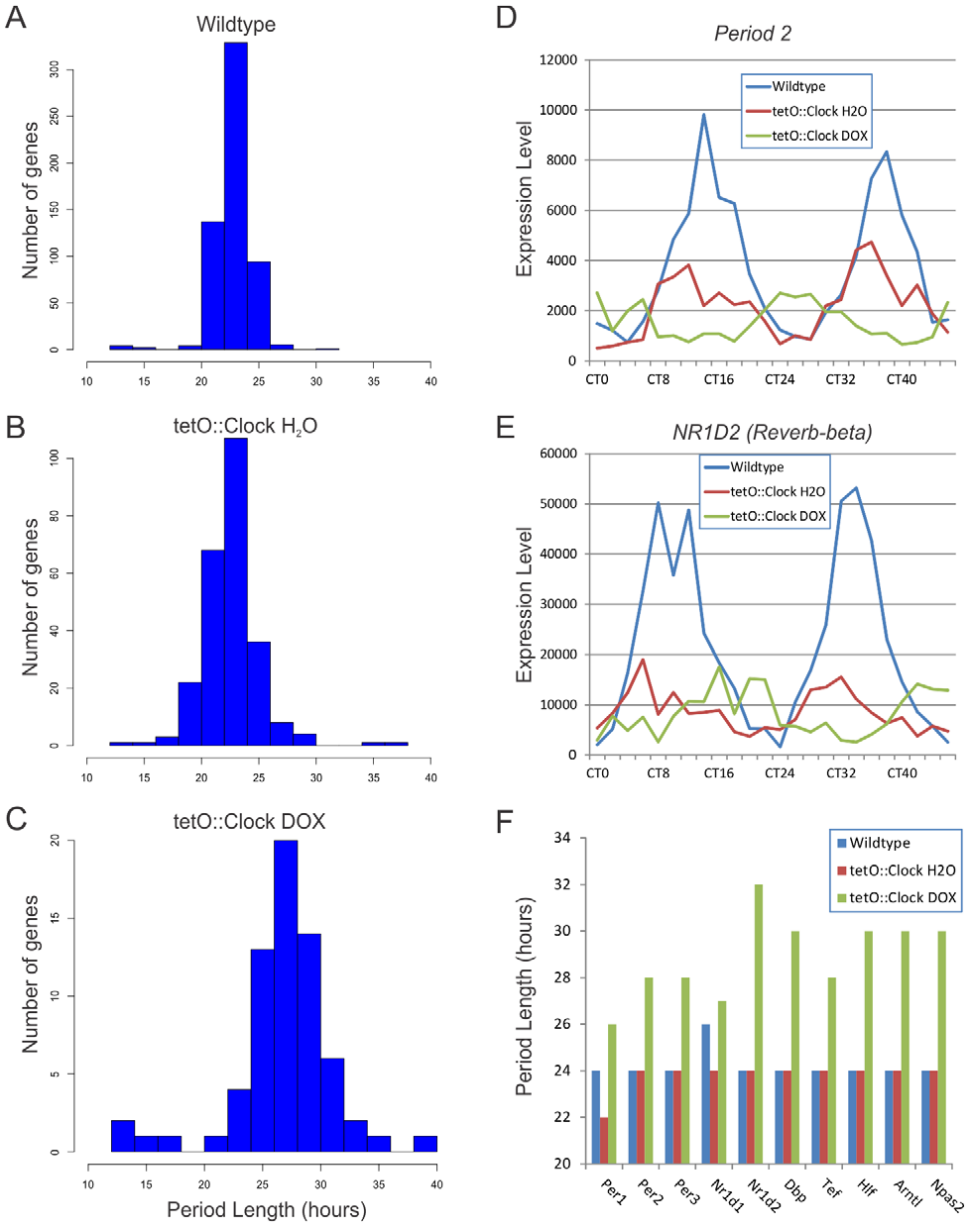
*Clock*-rescue restores ∼24-hour transcriptional rhythms. Panels A–C show histograms of the period lengths of cycling genes in wildtype (A), *Clock*-rescue (B) and *Clock*-mutant (C) livers (p<0.0011 for each genotype/treatment, corresponding to a q<0.05 in wildtype). *Clock*-mutant mice have long transcriptional rhythms with periods approximating their behavioral rhythms (mean period = 27.6+/−4.5 h), while wildtype and *Clock*-rescue animals have period lengths of approximately 24-hours (23.7+/−1.7 h and 23.6+/−2.6 h, respectively). Note that the difference in y-axis scales reflects the overall level of rhythmic transcriptional output in these mice. Two components of the core circadian clock, *Per2* (D) and *Nr1d2* (E), illustrate the long-period phenotype of the *Clock*-mutant mice. This effect is seen in most core clock genes as well as key circadian output genes (F). Additional examples are shown in Figure S1. The apparent phase difference in *Clock*-mutant mice compared to wildtype/*Clock*-rescue mice is believed to be a consequence of their free-running period length phenotype.

As expected, the average period length of transcriptional rhythms in wildtype mice was ∼24-hours (Figure 3A). Consistent with the behavioral profiles discussed above (Table 1), *Clock*-rescue mice (*tetO::Clock* H2O) also showed average period lengths of ∼24-hours, while *Clock*-defective mice (*tetO::Clock* DOX) had considerably longer period lengths as would be expected in *Clock^Δ19^* mutant mice (Figure 3B and 3C). This phenotype is illustrated by the profiles of two core clock genes, *Per2* and *Rev-erb-beta* (Figure 3D and 3E, and Figure S1). Both wildtype and *Clock*-rescue mice showed normal 24-hour rhythms with phases in agreement in both genotypes. In contrast, *Per2* and *Rev-erb-beta* in *Clock*-defective mice had a longer-period phenotype with peak expression out-of-phase with wildtype. Since these animals were housed in constant darkness for 2 days before sample collection, we believe this apparent phase difference is a consequence of their free-running period length phenotype. These observations were seen in every core clock gene tested, as well as many key circadian output genes (Figure 3F). In each case, these genes had ∼24-hour rhythms in wildtype and *Clock*-rescue mice, with considerably longer period length in *Clock*-defective mice.

Overall, 95 genes were found to cycle in both wildtype and *Clock*-rescue animals (p<0.0011, corresponding to a q<0.05 in wildtype). Three of these genes had period lengths of ∼12-hours in wildtype animals (i.e. circadian harmonics) and are discussed in greater detail below. The remaining 92 genes all showed period lengths of ∼24-hours in both wildtype and rescue animals, and 77 of the 92 (84%) have been previously seen to oscillate in mouse liver (Table 2) [19].

**Table 2:** *Clock*-rescue restores 24-hour rhythmicity to 92 circadian genes.

	Wildtype					tetO::Clock H2O (rescue)					24-hour rhythm in Hughes et al. 2009?	Systemically-driven in Kornmann et al. 2007?	Systemically-driven in JTK_Cycle re-analysis of Kornmann et al. 2007?	Reverb-alpha target in Le Martelot et al.?	Bmal target in Rey et al. 2011?
Gene	q-value	p-value	Period	Phase (hours)	Amplitude	q-value	p-value	Period	Phase (hours)	Amplitude					
0610010D20Rik	0.043499	0.001074	26	9	1.3	0.019096	3.60E-05	26	7	1.7	YES				
1110067D22Rik	0.000239	7.88E-07	24	22	4.1	0.005596	4.80E-06	24	21	1.5	YES				
5630401D24Rik	0.035929	0.00079	26	10	1.3	0.08455	0.00079	26	11	1.4					
A630005I04Rik	0.000239	7.88E-07	24	22	10.6	0.04346	0.000216	22	21	1.8	YES				
Abcb11	0.017929	0.000302	24	2	1.8	0.051058	0.000302	21	4.5	1.2	YES				
Abcg5	0.000334	1.26E-06	22	11	2.8	0.063073	0.000419	26	6	1.5	YES				
Abcg8	0.006556	7.56E-05	24	9	4.5	0.022227	5.24E-05	26	6	1.9	YES				
Adarb1	0.003737	3.03E-05	24	12.5	2.0	0.08455	0.00079	24	12	1.3					YES
Adora1	7.02E-05	1.05E-07	26	9	1.8	0.08455	0.00079	26	9	1.4	YES				
Agpat1	0.035929	0.00079	22	15	1.5	0.019096	3.60E-05	26	12	1.5					
Agtr1a	0.017929	0.000302	22	6	1.9	0.037685	0.000153	24	4	1.3	YES				
AI842396	0.006556	7.56E-05	23	6.5	1.7	0.073204	0.000577	24	6	1.5	YES				
Alas1	0.002233	1.66E-05	24	14	3.2	0.035038	0.000108	24	12	5.6	YES		YES		
Alpl	4.93E-05	6.13E-08	22	17	1.9	0.08455	0.00079	24	15	1.5					
Ankhd1	0.000175	4.87E-07	26	15	3.2	0.08455	0.00079	24	13.5	1.7	YES				
Arl4a	0.010948	0.000153	24	22	2.1	0.019096	3.60E-05	22	2	1.3	YES				
Arntl	4.93E-05	6.13E-08	24	23	34.5	0.019096	3.60E-05	24	21	2.2	YES			YES	YES
Atg2a	0.043499	0.001074	22	9	1.6	0.011737	1.11E-05	26	5	1.4					
Atp1a1	0.000903	4.80E-06	24	14.5	1.5	0.019096	3.60E-05	24	13	1.3	YES				
BC016495	0.000133	2.96E-07	26	14	3.3	0.004223	1.99E-06	28	14	2.0	YES				YES
Cabc1	0.000334	1.26E-06	24	1	4.0	0.011737	1.11E-05	22	4	1.8	YES		YES	YES	YES
Camk1d	0.00861	0.000108	24	0	2.4	0.04346	0.000216	22	2	2.3	YES				
Ccng2	0.010948	0.000153	24	22	2.3	0.04346	0.000216	24	0	1.7	YES				
Ccrn4l	0.00046	1.99E-06	24	14	4.1	0.08455	0.00079	22	13	7.4	YES	YES			YES
Celsr1	0.00046	1.99E-06	24	21	2.3	0.04346	0.000216	24	18	1.9	YES				
Chkb	0.005073	5.24E-05	22	4	1.6	0.037685	0.000153	22	3	1.4	YES				
Clpx	1.38E-05	5.92E-09	24	23	7.4	0.001228	6.13E-08	24	23	2.0	YES			YES	
Coq10b	0.000334	1.26E-06	24	13	3.9	0.04346	0.000216	24	13	2.7					YES
Crot	0.000175	4.87E-07	22	5	2.7	0.022227	5.24E-05	22	5	1.6	YES				
Dapk1	0.003837	3.60E-05	24	23	2.6	0.08455	0.00079	22	21	1.5	YES				YES
Dbp	7.02E-05	1.05E-07	24	11	10.7	0.04346	0.000216	24	9	1.6	YES		YES		YES
Enpp2	0.003061	2.45E-05	24	13	1.8	0.08455	0.00079	22	11	1.4	YES				
Etnk2	0.000334	1.26E-06	24	12	1.5	0.099034	0.001074	24	11	1.9	YES				YES
Fbxo21	0.003837	3.60E-05	22	8	2.9	0.037685	0.000153	24	5	1.8	YES	YES			YES
Fkbp4	0.017929	0.000302	24	19	1.7	0.012876	1.66E-05	24	16	1.7	YES				
Fmo5	0.000239	7.88E-07	22	17	2.1	0.04346	0.000216	24	15	1.5	YES			YES	YES
Gabarapl1	0.017929	0.000302	22	7	2.0	0.037685	0.000153	24	4	1.8	YES				
Gpt2	0.000175	4.87E-07	24	14	1.9	0.022227	5.24E-05	22	13	1.6	YES				YES
Hlf	0.000239	7.88E-07	24	14	2.3	0.004223	1.99E-06	24	13	1.6	YES				YES
Ivns1abp	0.028179	0.000577	24	14.5	1.6	0.063073	0.000419	24	13	1.4	YES				YES
Kcnk5	0.002233	1.66E-05	24	13	3.1	0.063073	0.000419	22	11	3.2	YES				
Klf15	0.006556	7.56E-05	24	12	1.5	0.051058	0.000302	24	12	1.6	YES				YES
Mad2l2	0.000175	4.87E-07	24	2	3.0	0.035038	0.000108	22	3	1.4	YES				
Man2a1	0.010948	0.000153	24	14	1.6	0.012876	1.66E-05	24	14	1.4					YES
Mapk14	0.001229	7.32E-06	24	16	1.6	0.08455	0.00079	24	14.5	1.3	YES				
Marveld1	1.96E-05	1.09E-08	24	15	2.1	0.016353	2.45E-05	24	13	1.7	YES				YES
Mfsd2	0.000903	4.80E-06	24	14	7.3	0.099034	0.001074	24	12.5	6.2	YES				
Mgrn1	0.001229	7.32E-06	24	10	1.5	0.073204	0.000577	28	5	1.3	YES				YES
Mov10	0.013737	0.000216	22	8	1.9	0.005596	4.80E-06	26	4	1.9					
Mpzl1	0.001624	1.11E-05	24	0	2.6	0.073204	0.000577	22	2	1.4	YES				
Mreg	0.000133	2.96E-07	22	2	2.3	0.08455	0.00079	22	2	1.4					
Mthfr	1.38E-05	5.92E-09	24	23	3.3	0.099034	0.001074	24	20	1.2	YES				
Mtss1	0.00861	0.000108	24	12	2.1	0.001384	1.78E-07	24	13	2.6	YES				YES
Ndrg1	0.000133	2.96E-07	24	2.5	2.4	0.063073	0.000419	20	5	2.3	YES			YES	
Npas2	3.72E-05	3.51E-08	24	1	2.7	0.037685	0.000153	24	22	3.3	YES				
Nr1d1	4.93E-05	6.13E-08	26	7	12.3	0.037685	0.000153	24	6	1.7	YES			YES	YES
Nr1d2	1.96E-05	1.09E-08	24	10.5	11.3	0.037685	0.000153	24	8	2.7	YES		YES		YES
P2rx4	0.013737	0.000216	23	10.5	1.7	0.063073	0.000419	28	4	1.6	YES				
Park2	0.000631	3.11E-06	26	8	1.5	0.003676	1.26E-06	26	7	1.7					
Per1	0.000133	2.96E-07	24	13	4.3	0.022227	5.24E-05	22	12	3.7	YES				YES
Per2	4.78E-06	4.10E-10	24	15	5.9	0.005596	4.80E-06	24	13	5.0	YES	YES			YES
Per3	0.000175	4.87E-07	24	13	11.2	0.003676	7.88E-07	24	12.5	2.5	YES				YES
Pex26	0.003061	2.45E-05	26	12	1.6	0.037685	0.000153	24	12.5	1.3	YES				YES
Pik3ap1	0.010948	0.000153	24	8	2.1	0.022227	5.24E-05	24	9	1.5	YES				YES
Pla2g12a	0.006556	7.56E-05	22	11	2.3	0.073204	0.000577	24	8	1.6	YES				YES
Por	6.35E-06	1.63E-09	24	14	3.5	0.04346	0.000216	24	13	1.6	YES			YES	
Ppp1r3b	0.013737	0.000216	24	18.5	3.8	0.051058	0.000302	24	14	1.9	YES				YES
Rhbdd2	0.000334	1.26E-06	24	14	2.3	0.016353	2.45E-05	26	11	1.4	YES				
Rnf125	0.010948	0.000153	22	2	4.9	0.003676	1.26E-06	22	2	4.4	YES				YES
Scap	0.00046	1.99E-06	22	8	1.6	0.08455	0.00079	26	3	1.5	YES				YES
Serpinf2	0.013737	0.000216	22	7	1.4	0.063073	0.000419	24	5	1.4	YES				
Sh3bp2	0.043499	0.001074	26	8	1.2	0.099034	0.001074	28	6	1.3	YES				
Slc17a3	0.017929	0.000302	24	21	2.1	0.037685	0.000153	26	16	1.7	YES				YES
Slc37a4	0.000175	4.87E-07	24	14	1.7	0.037685	0.000153	22	13	2.5	YES				
Slc5a6	0.00861	0.000108	24	11	2.1	0.051058	0.000302	24	11	1.5	YES				YES
Spon2	6.48E-05	8.33E-08	22	3	2.9	0.005578	3.11E-06	24	1	2.0	YES				
Srm	0.005073	5.24E-05	22	16	1.8	0.035038	0.000108	24	13.5	2.2	YES				YES
St3gal5	0.000631	3.11E-06	24	14.5	3.2	0.028443	7.56E-05	24	11	2.4	YES				YES
St5	0.001624	1.11E-05	24	23	5.6	0.022227	5.24E-05	22	0	1.7					
Syt1	0.013737	0.000216	24	9	3.4	0.001728	2.96E-07	24	6	1.9	YES				
Tef	3.72E-05	3.51E-08	24	12	7.0	0.005578	3.11E-06	24	13	2.0	YES				YES
Tmem218	0.001624	1.11E-05	26	1	3.1	0.001228	1.05E-07	22	2	1.8				YES	
Tns1	0.02232	0.000419	22	15	1.8	0.012876	1.66E-05	24	12	1.5					YES
Tsc22d3	0.002233	1.66E-05	24	17	2.6	0.063073	0.000419	22	13	3.1	YES			YES	YES
Ubxn1	0.000175	4.87E-07	22	5	2.0	0.012876	1.66E-05	22	5	1.6				YES	
Ulk1	0.010948	0.000153	22	9	1.9	0.08455	0.00079	24	4	1.2	YES				
Usp2	0.000631	3.11E-06	24	13	15.9	0.019096	3.60E-05	24	13	4.5	YES				YES
Wbscr27	0.00861	0.000108	24	5	1.8	0.037685	0.000153	24	4	1.5	YES				
Wdr45	0.010948	0.000153	22	9	1.5	0.051058	0.000302	24	6	1.7	YES				
Wrnip1	0.006556	7.56E-05	24	0	1.8	0.005596	4.80E-06	22	3	1.6	YES				YES
Ypel2	0.017929	0.000302	24	22.5	3.6	0.022227	5.24E-05	22	2	2.8					
Zfp295	9.88E-05	1.78E-07	24	21	1.7	0.028443	7.56E-05	24	17	1.4	YES				

**Figure 4:**
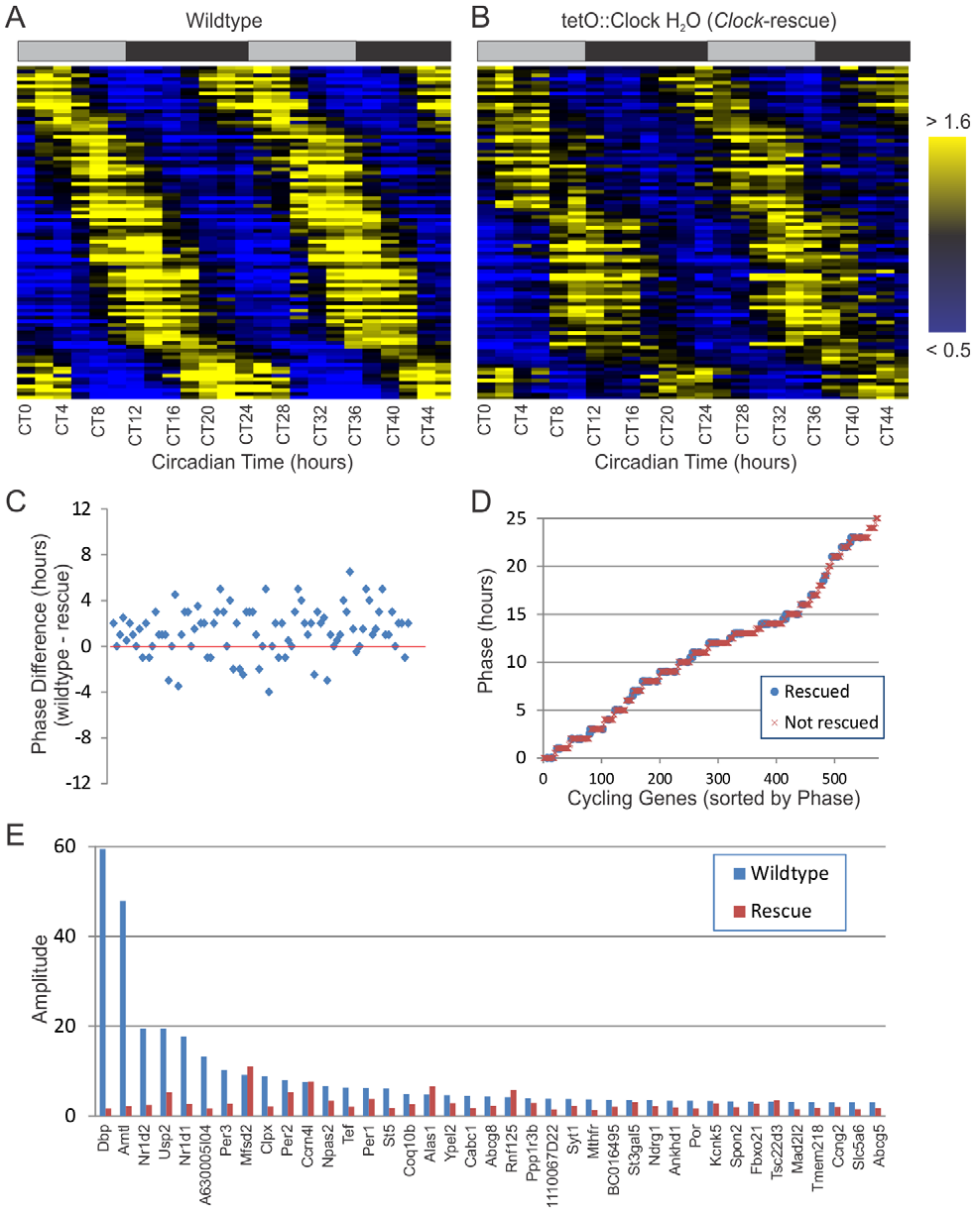
*Clock*-rescue restores appropriate phase to circadian output genes. 24-hour cycling genes (p<0.0011 for both genotypes, period ≥20 hours, N = 92) identified in both wildtype (A) and *Clock*-rescue animals (B) were median-normalized, sorted by phase, and plotted as a heatmap (yellow = high expression, blue = low expression). Each row represents the temporal expression of one cycling gene common to both datasets. Shaded bars above the heatmaps represent subjective day and night. In (C), the phase difference between wildtype and Clock-rescue is plotted for all 92 rescued genes, ordered by amplitude in wildtype. On average, there is a ∼1.5 h phase advance in the Clock-rescue samples. Panel (D) shows the phases of every cycling gene identified in wildtype, including those rescued in Clock-rescue animals (blue circles) and those not rescued (red x's). There was no bias in the phase of rescued genes. Panel (E) shows the amplitudes of high-amplitude cycling genes (AMP>3.0) in wildtype versus *Clock*-rescue animals. Several genes have been re-plotted from Figure 2E for the sake of completeness.

The phase of circadian output rhythms was restored by brain-specific *Clock*-rescue. The heatmaps in Figure 4A and 4B demonstrate striking similarity between the profiles of the circadian transcriptomes in both sets of animals. Figure 4C shows the phase difference between wildtype and Clock-rescue as a scatter plot for each of the 92 rescued circadian genes. These data points were centered near zero, although there was a modest (∼1.5-hour) phase-advance in *Clock*-rescue versus wildtype. This phase difference was less than one standard deviation from zero, so we do not consider it to be statistically significant, although we note that the slightly faster behavioral rhythms in *Clock*-rescue animals may account for this modest phase advance (Figure 1 and Table 1). Figure 4D shows the phases of all rescued (blue circles) and non-rescued genes (red x's). Rescued genes were found with peak expression at every time of day, and there was no significant bias in the phase of rescued versus non-rescued genes (chi-squared test).

Overall, 76 of 92 rescued genes (82%) had lower amplitudes in *Clock*-rescue animals versus wildtype, and the median amplitude in *Clock*-rescue was 20% lower than in wildtype. As would be expected given the un-rescued expression of *Clock^Δ19^* in the liver, the most significantly affected genes were high-amplitude cyclers that are direct targets of BMAL1/CLOCK. We also compared this dataset to known *Bmal1* and *Reverb-alpha* target genes (Table 2) [49], [50]. For example, *Dbp*, *Bmal1* (*Arntl*), *Rever-alpha* (*Nr1d1*), *Reverb-beta* (*Nr1d2*), *Per3*, and *Tef* are among the cycling genes with the most significant amplitude defect in rescue animals (Figure 4E). Likewise, *Ubiquitin Specific Protease 2* (*Usp2*) has significantly diminished amplitude in the rescue, and has also been shown to modify circadian rhythms [51] and is a direct target of CLOCK [52]. Taken as a whole, these data indicate that brain-specific rescue of *Clock* function is sufficient to restore normal period lengths and phases to a significant fraction of peripheral circadian output. However, the generation of robust circadian output (as measured by the number of cycling genes and their amplitude) depends on an intact peripheral oscillator.

Core clock genes were preferentially rescued by *Clock* expression in the SCN. We performed DAVID analysis to determine whether rescued genes represented specific pathways or ontologies [53]. We found that core clock genes were the only ontological group significantly enriched in this data set (n = 92, enrichment = 0.79, p<0.0005, q<0.07). Consistent with this observation, we found that core clock genes were preferentially rescued, even compared to other high-amplitude cycling genes (amplitude >5.0 peak∶trough). Of the 11 high-amplitude core clock genes, 9 showed normal rhythms in *Clock*-rescue mice. In contrast, only 7 of 28 high-amplitude output genes were rescued in these samples. Taken together, we conclude that key components of the circadian clock are sensitive to either direct or indirect signals from the SCN, even in the absence of a functional local circadian clock.

**Figure 5:**
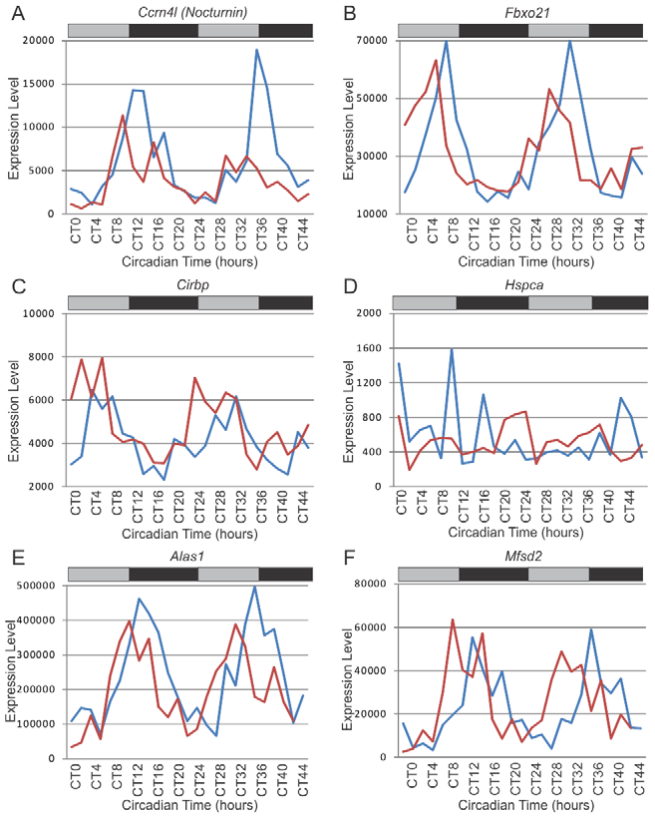
Examples of System-driven circadian transcripts. Microarray expression levels in wildtype (blue) and *Clock*-rescue (red) animals for six notable system-driven genes, including *Nocturnin* (A), *Fbxo21* (B), *Cirbp* (C), *Hspca* (D), *Alas1* (E), and *Mfsd2* (F). Grey and black bars represent subjective day and night, respectively.

Taking this line of investigation one step further, a direct comparison between our 92 rescued circadian genes and the 31 system-driven genes identified by Kornmann et al. [21] is of obvious importance. Surprisingly, there was very little overlap between these two data sets (Table 2). Only three genes were common to both sets: *Per2* (Figure 3D), *Nocturnin* (*Ccrn41*) (Figure 5A), and *Fbxo21* (Figure 5B). We reasoned that the apparent disagreement between these two data sets may be a consequence of inconsistencies in the underlying statistical analyses. To address this, we re-analyzed Kornmann et al.'s data using JTK_Cycle (Table S3). At the same statistical threshold used in the present study (p<0.0011), we found only three genes that were systemically-driven (*Ccdc12*, *Cry1*, and *Dbp*), none of which were previously identified. In the interest of identifying as many similarities as possible between the present study and Kornmann et al.'s data, we therefore loosened the statistical threshold to p<0.1, which corresponds to a FDR of q<0.41 in the wildtype (non-*Reverb-alpha* over-expresssing) condition. At this confidence level, we found 47 unique genes that were systemically-driven (Table S3), including five that overlap with Kornmann et al. (*1200016E24Rik*, *4833417J20Rik*, *Fus*, *Hsap1b*, *Tuba4*) and four that overlap with the present study (*Alas1*, *Cabc1*, *Dbp*, and *Nr1d2*).

Therefore, the apparent disagreement between the present study and Kornmann et al. can be partially reconciled by standardizing the statistical analyses. For example, *cold inducible RNA binding protein* (*Cirbp*) was identified and validated by Kornmann et al. as a system-driven gene. Although we do not detect it as being rhythmic in the *Clock*-rescue condition, if we loosen our statistical threshold, it is clearly rhythmic in both wildtype and *Clock*-rescue (Figure 5C). Further liberalizing statistical criteria can identify additional similarities between these studies, albeit at the expense of considerably more false-discoveries. Nevertheless, some genes are clearly divergent between these datasets, such as *Hspca* which is system-driven in Kornmann et al. but arrhythmic in our data (Figure 5D).

We speculate that these differences are due to the different genetic manipulations used in these studies. Kornmann et al. over-expressed *Reverb-alpha* to systematically inhibit *Bmal1*-mediated transcription, as well as every other *Reverb-alpha* target gene. Similarly, our study used *Clock^Δ19^* as a dominant mutant to knock-down *Bmal1*/*Clock* activity. However, *Clock^Δ19^* is not expected to dramatically affect *Reverb-alpha* target genes, which is supported by the presence of 10 *Reverb-alpha* targets among the 92 rescued circadian genes (Table 2) [50]. Moreover, *Clock^Δ19^* is insufficient to abolish all circadian molecular oscillations, as evidenced by the weak, long-period transcriptional rhythms seen in *Clock*-mutant mice. For both these reasons, we expected to see rescued transcriptional rhythms not previously seen in Kornmann et al. These rescued transcriptional rhythms could be bona fide system-driven genes, or downstream genes driven by the residual activity of the molecular oscillator in *Clock^Δ19^*, or some combination thereof.

Two candidate system-driven genes are shown in Figure 5E and 5F. *Aminolevulinic acid synthase 1* (*Alas1*) was not identified by Kornmann et al., but was detected as system-driven in our re-analysis of their dataset (Table S3). It oscillates with normal period and phase in both wildtype and *Clock*-rescue, and has an amplitude approximately the same in both genotypes/treatments, as would be expected from a system-driven gene (Figure 4E). Interestingly, *Alas1* forms a junction between the circadian clock and heme bioactivity. It is the rate-limiting enzyme in heme biosynthesis and is regulated by Npas2. Additionally, it regulates the activity of Bmal1/Npas2, ultimately affecting the expression of *Per1* and *Per2* [54], thereby making it a strong candidate for conveying system-driven cues into the peripheral circadian clock.

Similarly, *major facilitator superfamily domain containing 2A* (*Mfsd2*) is rhythmic in *Clock-*rescue animals (Figure 5F). Like other potential system-driven genes (e.g. *Per2* and *Nocturnin*), *Mfsd2* expression is largely unchanged between wildtype and *Clock*-rescue animals (Figure 4E). Interestingly, *Mfsd2* is highly induced in liver and brown fat by fasting and cold-induced thermogenesis [55], consistent with (and a possible molecular mechanism for) Kornmann et al.'s hypothesis that temperature is a major entrainer of peripheral circadian clocks. At the least, *Mfsd2* is an excellent candidate for conveying nutritional signals to the liver clock.

In addition to 24-hour transcriptional rhythms, the liver and other tissues express ultradian rhythms with period lengths of 12- and 8- hours [19]. A recent study has demonstrated that at least some of these circadian ‘harmonics’ are disorganized in mice with genetically-disrupted circadian oscillations [56]. However, the extent to which these rhythms are driven by central versus peripheral oscillators is unclear.

**Figure 6:**
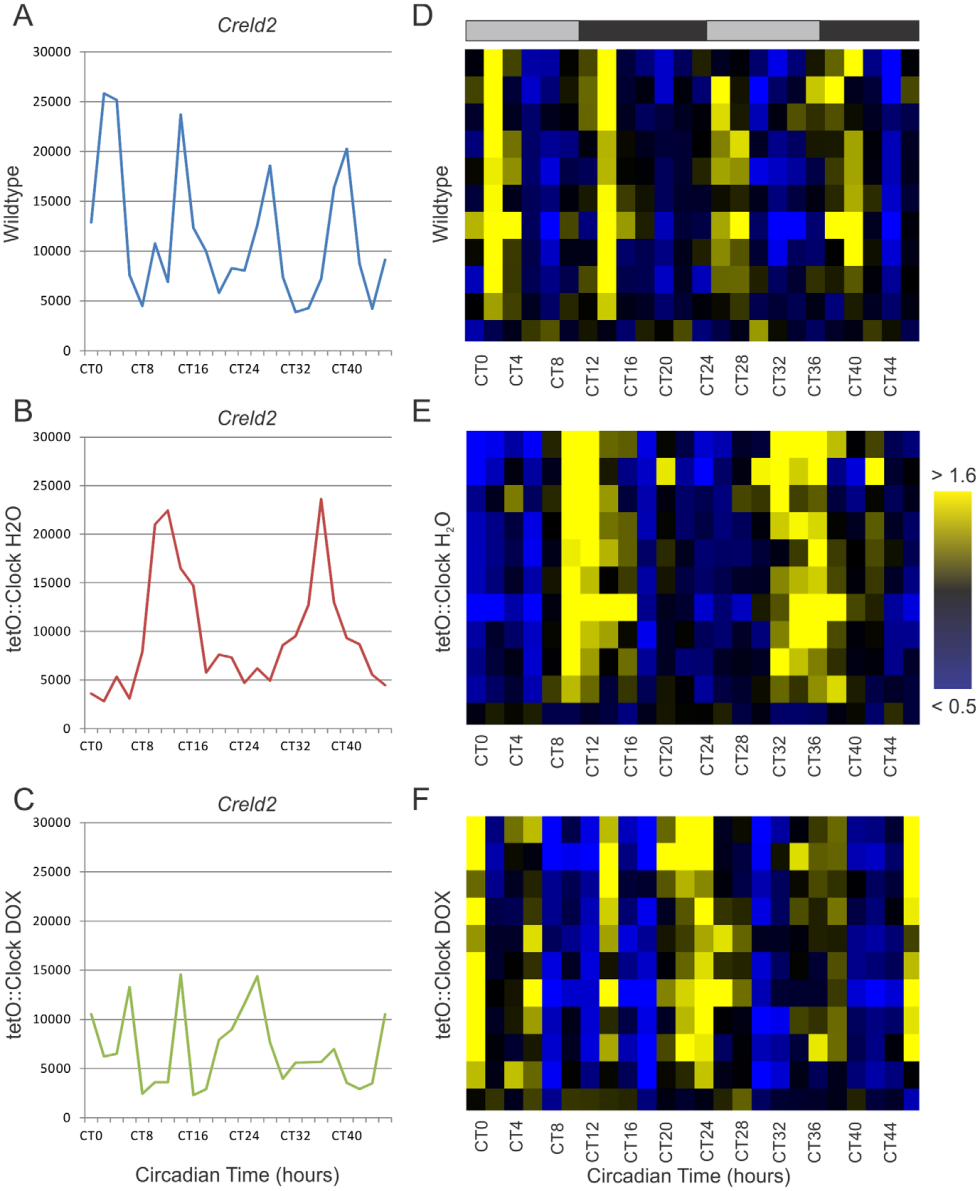
*Clock*-rescue restores 24- but not 12-hour rhythms to circadian harmonics. Panels A–C show the time course of *Creld2* expression (a known 12-hour cycling gene) in wildtype (A), *Clock*-rescue (B) and *Clock*-mutant (C) livers. Panels D–F show a subset (N = 11) of wildtype 12-hour rhythms median-normalized and plotted as a heatmap (yellow = high expression, blue = low expression). Every line represents the temporal expression of one wildtype 12-hour cycling gene. Shaded bars above the heatmaps represent subjective day and night.

**Figure 7:**
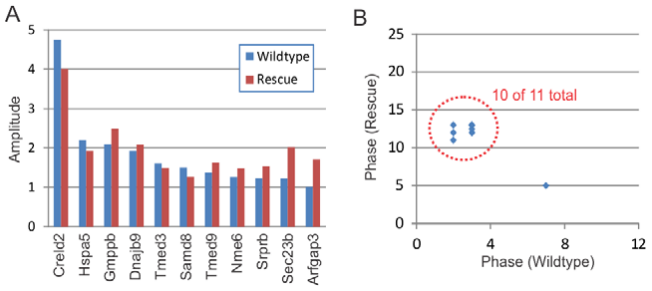
Clock-rescue restores normal amplitude and phase to circadian harmonics. Panel (A) shows the amplitude of circadian harmonics in wildtype (blue) and *Clock*-rescue (red) animals. In every case, there was no obvious diminishment of amplitude in the rescue animals. Panel (B) plots the phase of these rhythms in wildtype (x-axis) versus *Clock*-rescue (y-axis). 10 of the 11 total genes had similar phases in both genotypes.

To address this, we examined the transcriptional profiles of circadian harmonics in wildtype, *Clock*-rescue, and *Clock*-defective mice. For example, *Creld2* was previously identified as a 12-hour oscillator [19], and re-confirmed by this study (Figure 6A). This ultradian oscillator reverted to a 24-hour period length in the *Clock*-rescue mice (Figure 6B), and became disorganized with lower overall expression in *Clock*-defective mice (Figure 6C). This phenotype is consistent with the other 12-hour rhythms detected in these data (N = 3, Table 3), as well as previously identified 12-hour cyclers [19] with some evidence of oscillatory behavior (period = ∼12-hours AND p<0.1) in the present data set. In the every case, 12-hour oscillations revert to 24-hour period lengths in *Clock*-rescue mice (Figure 6D and 6E, and Table 3). Interestingly, the disorganization of these rhythms in *Clock*-defective mice is consistent between genes (Figure 6F), strongly suggesting that their promoters share common transcriptional inputs. Moreover, the amplitudes of normal 12-hour rhythms and rescued 24-hour rhythms are largely indistinguishable (Figure 7A), indicating that at least one of the two daily peaks of expression in wildtype is largely driven by systemic cues and not the local, peripheral oscillator. Likewise, the phases of the normal and rescued rhythms largely fall into a single cluster (Figure 7B), consistent with the idea that they are responding to identical circulating cues. Given the unambiguous defect in generating 12-hour rhythms in *Clock*-rescue animals, we conclude that the local, peripheral oscillator is absolutely required for generating 12-hour transcriptional rhythms. However, the appearance of 24-hours in the absence of an intact liver clock suggests that half the peaks of these oscillations are derived from circulating cues downstream of the central oscillator in the SCN.

**Table 3:** 12-hour transcriptional rhythms revert to 24-hour rhythms when *Clock* is driven in the SCN.

		Wildtype			tetO::Clock H2O			tetO::Clock DOX		
Probe Set ID	Gene	p-value (FDR)	Period (hours)	Phase (hours)	p-value (FDR)	Period (hours)	Phase (hours)	p-value (FDR)	Period (hours)	Phase (hours)
6837437	Arfgap3	0.027 (0.45)	12	2.0	0.034 (0.61)	22	11.0	1.00 (1.0)	28.4	16.9
6800427	Dnajb9	0.003 (0.08)	12	2.0	0.286 (1.0)	20	12.0	1.00 (1.0)	28.4	17.5
6992306	Gmppb	3.02 E-04 (0.02)	12	2.0	7.56 E-05 (0.03)	24	13.0	1.00 (1.0)	28.4	15.1
6997671	Tmed3	0.043 (0.61)	12	3.0	0.003 (0.18)	24	12.0	0.193 (1.0)	12	1.0
6992409	Nme6	0.054 (0.71)	12	3.0	0.001 (0.10)	24	12.0	1.00 (1.0)	28.4	15.5
6998396	Srprb	0.003 (0.10)	12	3.0	2.16 E-04 (0.04)	24	12.5	1.00 (1.0)	28.4	16.4
6832530	Creld2	0.010 (0.22)	12	3.0	1.26 E-06 (0.004)	24	13.0	1.00 (1.0)	28.4	16.1
6876342	Hspa5	0.001 (0.04)	12	3.0	1.53 E-04 (0.38)	24	13.0	0.235 (1.0)	30	27.0
6807251	Tmed9	0.068 (0.82)	12	3.0	0.006 (0.24)	24	13.0	1.00 (1.0)	28.4	15.1
6881837	Sec23b	2.45 E-05 (0.003)	12	3.0	0.001 (0.10)	24	13.0	0.416 (1.0)	12	0.0
6817412	Samd8	0.005 (0.12)	12	7.0	0.104 (1.0)	20	5.0	1.00 (1.0)	28.4	9.4

## Discussion

The circadian regulatory network is organized in a hierarchical fashion with signals originating in the SCN orchestrating rhythms in peripheral tissues. Understanding the nature of these signals and how they are communicated to peripheral tissues is a significant challenge for the field [57]. Here, we have used the tet-OFF expression system to rescue normal circadian function in the brains of otherwise circadian-defective mice. This approach allowed us to dissect aspects of peripheral rhythms that depend on central oscillator function from those that depend on local circadian oscillators.

Using microarray analysis to characterize the circadian transcriptome of wildtype, *Clock*-rescue, and *Clock*-mutant mice, we found that brain-specific rescue of *Clock* partially restores circadian transcriptional output in the liver. The number of cycling genes was significantly higher in *Clock*-rescue mice compared to *Clock*-defective mice (Figure 2B). Likewise, the confidence with which core clock genes were identified as cycling was greater in the rescue animals (Figure 2D). Both period length (Figure 3) and phase (Figure 4) of transcriptional rhythms in the liver of *Clock*-rescue mice were consistent with their wildtype counterparts. In contrast, rhythms in *Clock*-defective mice were out-of-phase with wildtype and had significantly longer period lengths. These results agree with studies of transplanted fibroblasts that have been shown to synchronize their local circadian oscillators to the SCN of their host [58]. Taken as whole, these data indicate that signaling downstream of the central circadian oscillator is sufficient to reset the period and phase of many peripheral transcriptional rhythms in tissues that otherwise have disrupted circadian oscillations.

Nevertheless, the total amount of circadian transcriptional output (as measured by the number of cycling genes and their amplitudes) was still significantly less than wildtype (see Figure 2B and 2E, Figure 3D and 3E, and Figure 4). This observation is consistent with a recent study by Kornmann et al. that profiled the liver circadian transcriptome in *ad libitum* fed mice with disrupted circadian oscillations in the liver due to the over-expression of Rev-erb-alpha [21]. In that study, ∼10% of cycling transcripts (31 of 351) continued to oscillate with normal periods and phases independently of the genetic manipulation to their peripheral clock, although the amplitudes of these rhythms were generally diminished. Similarly, we saw 16.5% (952 of 576) of genes had restored oscillations in *Clock*-rescue animals at a threshold of p<0.0011, corresponding to a FDR of q<0.05 (Figure 2C).

Unlike the Kornmann et al. study, however, we detected 24-hour oscillations of many key circadian genes in *Clock*-rescue animals, including *Nr1d1*, *Arntl*, *Per1/Per2/Per3*, and *Dbp/Tef/Hlf* (Table 2). This disagreement between the present study and Kornmann et al. may not be as dramatic as it initially appears. When re-analzying the Kornmann et al. dataset with JTK_Cycle, we found *Cry1*, *Dbp*, and *Nr1d2* to all be system-driven. We therefore conclude that 24-hour periodicity in many components of the core circadian clock can be driven by oscillations in the SCN. The extent to which this rescue of peripheral clock periodicity is due to restored behavioral rhythmicity, humoral cues, or their interaction is unknown.

We noticed that core clock genes were preferentially rescued by restoring CNS clock function. This held true even when compared with other high amplitude oscillating genes in the liver. We speculate that the promoters of clock genes may have evolved configurations of response elements that are particularly sensitive to humoral and behavioral cues. One can imagine that this property – sensitivity to the CNS clock – would be particularly advantageous for resetting to different light schedules. Core clock gene action, subsequently, would then synchronize tissue specific peripheral gene expression appropriately.

At a broader level, we discovered 92 circadian genes (including clock factors) that have normal phases and periodicity in *Clock*-rescue animals. Since the rescue of *Clock* expression is largely confined to the brain, this restoration of normal phase and period is may be due to direct signaling from humoral cues emerging from the SCN, or alternatively due to indirect signaling, via the SCN's regulation of locomotor and feeding rhythms. In either case, a subset of rescued transcriptional rhythms is expected to be system-driven.

Kornmann et al. have previously identified 31 system-driven circadian genes by over-expressing *Reverb-alpha* in the liver [21]. Surprisingly, there were only three genes in common between their dataset and the present study. Even when using standardized statistical methods, only 7 of the 92 rescued genes identified herein were found in common with Kornmann et al. This is not entirely unexpected given the difference in the genetic manipulations used. For example, over-expressing Reverb-alpha would be expected to inhibit the expression of any genes under the control of ROR elements. Consistent with this idea, we found 10 rescued genes not previously identified by Kornmann et al. that are known targets of Reverb-alpha [50].

The genetic lesion used in this study, *Clock^Δ19^*, significantly diminishes the number of rhythmic genes as well as their amplitudes. However, some rhythmicity still persists, as evidenced by the weak, long-period rhythms still seen in *Clock*-mutant animals (Figure 2). At a mechanistic level, this implies that rescued genes in our dataset may be bona fide system-driven genes or peripheral clock-driven genes that are insensitive to CLOCK signaling. Consequently, strong candidates for system-driven genes such as Per2 and Nocturnin (identified by both Kornmann et al. and the present study) have amplitudes that are largely independent of genetic manipulation (Figure 4E). Based on this criteria, we identified two novel system-driven candidates: *Alas1* and *Mfsd2*. *Alas1* regulates and is regulated in turn by clock factors, while providing a link between the clock and heme biosynthesis [54]. Similarly, *Mfsd2* is known to be driven by the clock, and is upregulated by fasting and cold-induced thermogenesis [55]. Both genes are thus potential nodes through which systemic cues synchronize and drive peripheral circadian rhythms.

Both our study and Kornmann et al. agree that the number of system-driven circadian genes may be relatively high. Even accounting for the measurable false discovery rate of both studies, there are likely several dozen and potentially as many as a hundred cycling genes in the liver than can be driven in part by systemic cues. We speculate that this may be an evolutionary strategy to tightly link peripheral clocks with the physiological status of the animal. Unlike the SCN, peripheral clocks (especially the liver) are sensitive to a wide array of behavioral, environmental, and physiological stimuli. By having many different input pathways to synchronize liver rhythms, evolution may have built considerable redundancy into the system. This is analogous to the coupling of SCN neurons, which renders the entire timekeeping mechanism considerably more resistant to perturbation [59], [60].

Similarly to 24-hour rhythms, circadian harmonics (i.e., 12- and 8-hour rhythms), are transcriptional oscillations found in tissues throughout the body that persist in constant darkness [19], [56]. These rhythms have been confirmed to exist at the protein level, and may be a consequence of rhythms of lipid metabolism and ER stress [56]. Consistent with this observation, 12-hour transcriptional rhythms are disrupted in mice subjected to restricted feeding [19]. Nevertheless, the extent to which central and peripheral oscillators contribute to circadian harmonics is an open question.

We found that disrupting *Clock* function throughout the body disorganized and diminished 12-hour rhythms, indicating that these rhythms derive (at least in part) from the conventional 24-hour circadian clock (Figure 5 and Table 3). Interestingly, brain-specific rescue of *Clock* function restored 24- but not 12-hour rhythmicity to these genes, with no discernible loss of amplitude (Figure 6, Figure 7A, and Table 3). This observation is reminiscent of previous studies that demonstrated that both restricted feeding and *Hlf/Dbp/Tef* loss-of-function convert 12-hour rhythms to 24-hour period lengths [19], [56]. Moreover, the phase of 12-hour transcription is precisely the same regardless of which tissues are examined, suggesting a common signaling origin [19].

Since 24-hour periodicity of most clock genes is restored in the livers of *Clock*-rescue mice (albeit at consistently low amplitudes), we acknowledge that the rescue of 12-hour rhythms may be downstream of local oscillations in the liver. Nevertheless, we favor the explanation that circulating, tissue-non-autonomous signaling cues drive one of the two daily peaks of 12-hour transcriptional rhythms. Two observations support this interpretation. First, the amplitude of rescued circadian harmonics is largely the same as in wildtype (Figure 7B). In contrast, most core clock genes and key circadian outputs have low-amplitude oscillations in the rescued livers (Figure 4E and Table 2). We consider it more likely that systemic cues downstream of fully rescued SCN and the resulting behavioral rhythms drive these rescued harmonic oscillations rather than low-amplitude oscillations of clock genes in the liver. Second, previous studies in dissociated cells with normal molecular oscillations have consistently failed to detect harmonic transcriptional rhythms [19], [46]. Although these studies do not speak to the necessity of peripheral circadian clocks for generating 12-hour rhythms, they do indicate that cell-autonomous circadian oscillations are not sufficient for generating circadian harmonics. Regardless, based on the present study we can conclude for the first time that the conventional 24-hour circadian oscillator (whether central or peripheral) is the ultimate origin of harmonic transcriptional rhythms, and circulating cues downstream of the SCN are sufficient to restore one of the two daily peaks of expression.

## Materials and Methods

### Circadian tissue collection

Mice were housed in light-tight boxes and entrained to a 12 hour light, 12 hour dark schedule for one week before being switched to complete darkness. Wildtype mice (*C57BL/6J*) were acquired from Jackson Labs; experimental animals (*Scg2*::tTA; tetO::*Clock^wt^*; *Clock^Δ19/Δ19^*) were generated as previously described [36], [40]. The *Scg2*::tTA mice were congenic on a C57BL/6J background, and the tetO::*Clock^wt^* transgenic mice were co-isogenic on a C57BL/6J background. Mice were supplied with regular food and water *ad libitum*. Three days before the final lights off (at ZT12), experimental animals were treated with either water or doxycycline (30 ug/ml; Sigma-Aldrich) supplied in the drinking water. Starting two days after the first day in DD (i.e., at CT48), four wildtype mice (two females and two males) and *Clock*-rescue mice (one female and one male) were sacrificed in the dark every two hours. Liver samples were quickly excised and snap-frozen in liquid nitrogen. Liver samples were homogenized in Trizol (Invitrogen) and total RNA was purified using the manufacturer's protocol. All animal experiments were performed with the approval of the Committee on Animal Care and Use at Northwestern University.

### Circadian activity analysis

Mice were placed in individual running wheel cages and activity was recorded and analyzed using the ClockLab Data Collection System (Actimetrics, Wilmette, IL). Mice were entrained to a 12-hour light/12-hour dark cycle (LD) for a minimum of 10 days before they were released into constant darkness (DD). Doxycycline was supplied in the drinking water at a concentration of 10 µg/ml for the behavioral analysis as described previously [36], [40]. Mice were supplied with regular food and water with or without doxycycline *ad libitum*. Dox-containing water was renewed every 2–3 days.

### Quantitative PCR

RNA isolated from the liver were reverse transcribed with SuperScript First-Strand Synthesis System for RT-PCR (Invitrogen). cDNA (1.25 µl) was pre-amplified for specific target amplification (STA) using pooled 0.2× TaqMan Gene Expression Assays mix (Applied Biosystems). The thermal cycling conditions used for STA were 95°C hold for 10 minutes followed by 14 cycles of 95°C for 15 seconds and 60°C for 4 minutes. TaqMan Gene Expression Assays (Applied Biosystems) used in this study are listed in the supplemental data (Table S4). Preamplified cDNA was diluted with TE buffer (1∶5) and qPCR assays were performed using the BioMark 48.48 Dynamic Array as specified (Fluidigm). Assays were run in triplicate on each array and data were analyzed by the use of BioMark Real-Time PCR Analysis Software Version 2.0 (Fluidigm) to obtain Ct and *Δ* *Δ*Ct values. Quantitative PCR analysis (Figure S1) was performed from the same RNA samples as those used for the microarray analysis (see below).

### Microarray analysis

5 µg total RNA per time point was submitted to the University of Pennsylvania School of Medicine Microarray Facility for labeling and hybridization to Affymetrix Mouse Exon 1.0 ST Arrays. Expression values were extracted using RMA implemented in Expression Console (Affymetrix) at the core gene level. JTK_Cycle implemented in R (version 2.12.1, 64-bit) was used to detect cycling genes as previously described [37], using a period length window of 10–40 hours. Due to the lower sampling resolution, re-analysis of the Kornmann et al. 2009 dataset was performed using a 24 hour period length window. Raw data and statistics were compiled into an Access database (Microsoft). Heatmaps were generated using custom scripts implemented in MATLAB (Mathworks, version R2010b). DAVID analysis was performed as previously described [53], using all rhythmic genes in wildtype (N = 576, q<0.05, p<0.0011) as a background list, and all 24-hour rescued genes (n = 92) as the principal gene list. Amplitude estimates were made using JTK_Cycle, modified as previously described ((2*JTK.AMP)+(percentile(array, 0.1)/(percentile(array, 0.1)) [61] All .CEL files are available from GEO (accession number: GSE30411); custom scripts for MATLAB and R are available on demand.

## Supporting Information

Figure S1 qPCR validation of microarray data in core clock genes. Wildtype rhythms (top panels, solid blue lines, right axis) for *Per1* (A), *Per2* (B), *Per3* (C), *Arntl* (D), *Npas2* (E), and *Nr1d1* (F) show robust circadian oscillations with phases in close agreement with microarray data (grey dashed lines, left axis). Although lower amplitude in *Clock*-rescue animals (middle panels, red solid lines, right axis), these samples show appropriate period lengths and phases, in agreement with microarray data (grey dashed lines, left axis). In contrast, *Clock*-mutant mice (lower panels, green solid lines, right axis) show long-period rhythms out of phase with the wildtype samples and in agreement with microarray data (grey dashed lines, left axis). This apparent phase difference is believed to be a consequence of the free-running period length phenotype of *Clock*-mutant mice. Input RNA samples were the same for qPCR and microarray measurements and should therefore be considered technical replicates.
(TIF)

Figure S2 Cycling genes and their amplitudes. Microarray expression levels for circadian genes (p<0.0011, period ≥20 hours) in wildtype (A), *Clock*-rescue (C), and *Clock*-mutant (E) animals were median-normalized, sorted by phase, and plotted as a heatmap (yellow = high expression, blue = low expression). Shaded bars above the heatmaps represent subjective day and night. The amplitudes of these cycling genes are shown for wildtype (B), *Clock*-rescue (D), and *Clock*-mutant (F) animals (wildtype N = 570, *Clock*-rescue N = 248, *Clock*-mutant N = 63).
(TIF)

Table S1 JTK_Cycle statistics of the top cycling genes in wildtype, *tetO::Clock* H2O, and *tetO::Clock* DOX datasets (JTK_Cycle p-value<0.1; key circadian genes shown in bold).
(XLSX)

Table S2 Non-wildtype cycling genes. There are 187 genes that cycle (p<0.0011) in Clock-rescue and Clock-mutant animals but do not cycle in wildtype. Of these, 102 were previously found to oscillate in wildtype animals [19].
(XLSX)

Table S3 JTK_Cycle re-analysis of Kornmann et al. 2007 [21].
(XLSX)

Table S4 qPCR probsets used in Figure S1.
(XLSX)
