# Supplementary figures and images for: Brain-Specific Rescue of Clock Reveals System-Driven Transcriptional Rhythms in Peripheral Tissue
Source: PLoS Genet. 2012 Jul 26;8(7):e1002835. doi: 10.1371/journal.pgen.1002835 (PMC3405989; doi:10.1371/journal.pgen.1002835)

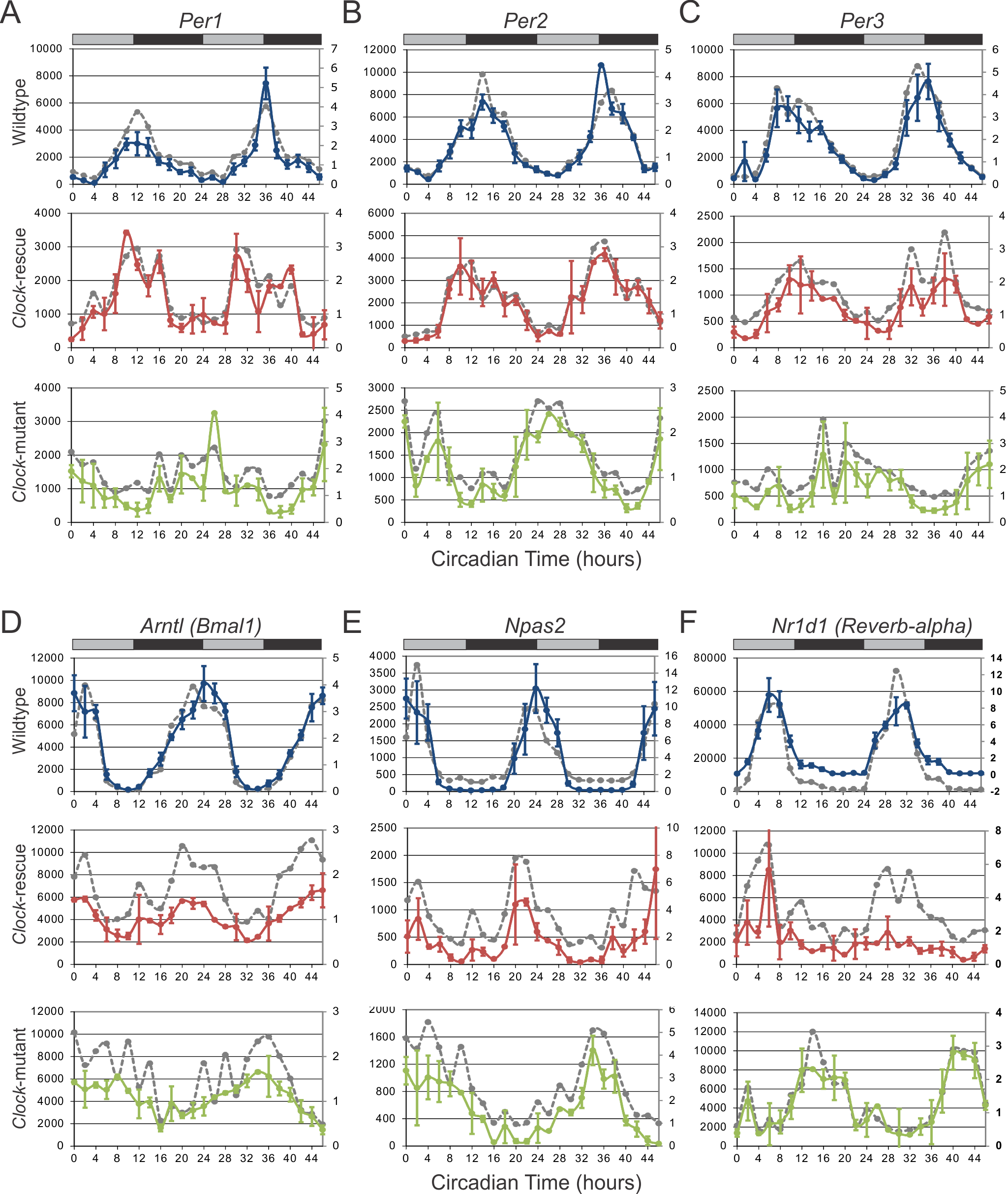

Supplement: Figure S1 — qPCR validation of microarray data in core clock genes. Wildtype rhythms (top panels, solid blue lines, right axis) for Per1 (A), Per2 (B), Per3 (C), Arntl (D), Npas2 (E), and Nr1d1 (F) show robust circadian oscillations with phases in close agreement with microarray data (grey dashed lines, left axis). Although lower amplitude in Clock-rescue animals (middle panels, red solid lines, right axis), these samples show appropriate period lengths and phases, in agreement with microarray data (grey dashed lines, left axis). In contrast, Clock-mutant mice (lower panels, green solid lines, right axis) show long-period rhythms out of phase with the wildtype samples and in agreement with microarray data (grey dashed lines, left axis). This apparent phase difference is believed to be a consequence of the free-running period length phenotype of Clock-mutant mice. Input RNA samples were the same for qPCR and microarray measurements and should therefore be considered technical replicates. (TIF) [file pgen.1002835.s001.tif]

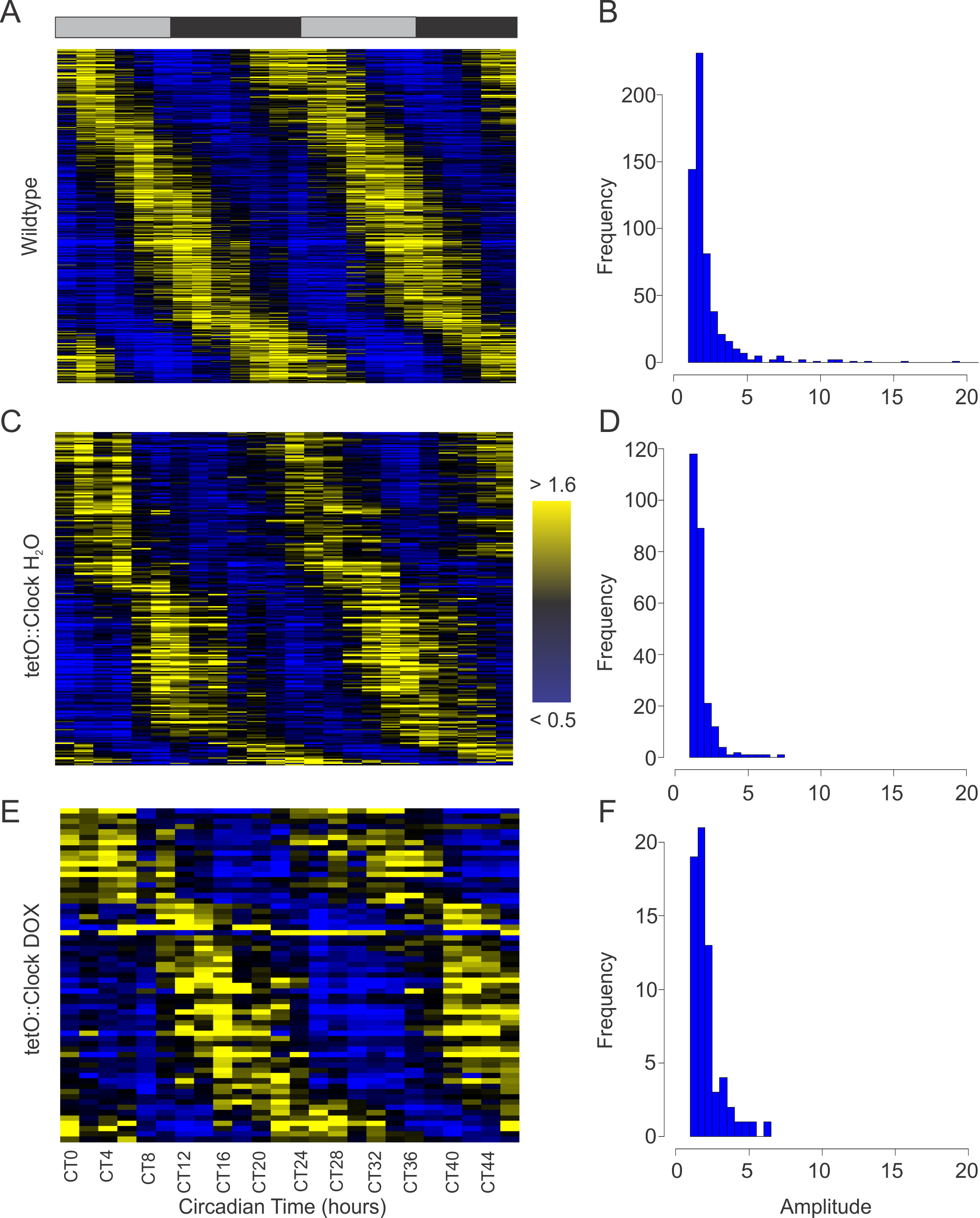

Supplement: Figure S2 — Cycling genes and their amplitudes. Microarray expression levels for circadian genes (p<0.0011, period ≥20 hours) in wildtype (A), Clock-rescue (C), and Clock-mutant (E) animals were median-normalized, sorted by phase, and plotted as a heatmap (yellow = high expression, blue = low expression). Shaded bars above the heatmaps represent subjective day and night. The amplitudes of these cycling genes are shown for wildtype (B), Clock-rescue (D), and Clock-mutant (F) animals (wildtype N = 570, Clock-rescue N = 248, Clock-mutant N = 63). (TIF) [file pgen.1002835.s002.tif]
